# Microbially mediated carbon utilization by a cold-water coral inhabiting methane seeps

**DOI:** 10.1038/s41598-025-32153-0

**Published:** 2026-02-24

**Authors:** April Stabbins, Shana Goffredi, Ryan Gasbarro, Katherine Dawson, John Magyar, Amanda Glazier, Kelly Meinert, Victoria Orphan, Erik Cordes

**Affiliations:** 1 Department of Biology, Temple University, Philadelphia, PA 19122 USA; 2 Department of Biology, Occidental College, Los Angeles, CA USA; 3 Department of Environmental Sciences, Rutgers University, New Brunswick, NJ USA; 4 Division of Geological and Planetary Sciences, California Institute of Technology, Pasadena, CA USA; 5 Prince William Sound College, University of Alaska, Valdez, AK USA; 6 Present Address: Institute of Marine Sciences, University of California, Santa Cruz, Santa Cruz, CA USA

**Keywords:** Chemosymbiosis, Deep sea, Corals, Microbiome, Habitat suitability modeling, Stable isotope analysis, Microbial ecology, Stable isotope analysis

## Abstract

Deep-sea methane seeps fuel biodiverse habitats sustained by the release of hydrocarbon-rich fluids and associated microbial activity. Here, we describe the ecology of a seep-associated cold-water coral and provide evidence of its associations with chemosynthetic bacteria. High-resolution seafloor surveys revealed that the distribution of this coral was predominantly confined to actively seeping zones, and habitat suitability models confirmed that proximity to active seepage was an important factor influencing the coral’s distribution. Stable carbon-isotope values were consistent with a nutritional strategy incorporating chemosynthetically derived carbon, likely as a supplement to suspension feeding on photosynthetically derived material. Microbial metabarcoding confirmed the presence of both thiotrophic and methanotrophic bacteria, including SUP05 and MMG-2 groups. Incubations with ^13^C-labelled methane further revealed this species may also be capable of assimilating methane-derived carbon into its biomass. These findings provide new evidence of a previously underrecognized facultative symbiosis between cold-water corals and chemosymbiotic bacteria and suggest that these corals are not restricted to the periphery of seep habitats. Instead, they may exploit microbial associations, including contributions from both thiotrophic and potentially methanotrophic taxa, to persist in actively seeping areas.

**Supplementary Information:**

The online version contains supplementary material available at 10.1038/s41598-025-32153-0.

## Introduction

Methane seeps occur along continental margins worldwide and host dense communities of unique organisms adapted to survive by forming relationships with or directly consuming chemosynthetic microbes to harness inorganic carbon. These microbes increase nutrient availability by obtaining energy from the oxidation of reduced gases such as hydrogen sulfide and methane as they are released from deep sediments. While most known chemosymbiotic relationships are formed between invertebrates and sulfide-oxidizing (thiotrophic) bacteria, some invertebrates are known to associate with methane-oxidizing (methanotrophic) bacteria, particularly in areas where high levels of methane reach the sediment–water interface^1–4^.

Symbiotic relationships with microbes, regardless of depth, have long been essential to the survival of multicellular marine organisms and have been crucial in shaping their ecology and evolution^5^. Animals from at least seven phyla are now recognised to form mutualistic symbiotic relationships with chemosynthetic bacteria^1,6^, but it was only recently that Cnidaria were added to this list^7,8^. Mutualisms between shallow, photoautotrophic Anthozoa and their symbionts are among the most extensively documented in marine ecology. The ecological success of this group after their rapid expansion and diversification during the Triassic period is likely due to the nutritional benefits gained from symbiosis with photosynthetic microalgae^9,10^. Given their common nutritional symbioses in shallow waters and their broad distributions across both depth and latitude, it is surprising that similar relationships between deep-sea Anthozoa and chemosynthetic microbes are only now becoming more apparent.

Although often overlooked, cold-water corals (CWC) comprise a highly diverse assemblage within Anthozoa, with more species found in deep-sea habitats than in shallower reef environments^11^. These CWC are ecologically important species, increasing habitat heterogeneity and supporting high levels of biodiversity by providing essential habitat for other organisms^12–15^. Their slow growth and recruitment rates make these species particularly vulnerable to anthropogenic disturbance, such as deep-sea mining, trawling, hydrocarbon extraction and climate change^16–24^.

Various CWC species have now been observed in chemically reducing habitats, often on the periphery where they take advantage of microbially precipitated authigenic carbonates as hard substrate, once seepage has subsided^13,25,26^. Less commonly, they can be observed within actively seeping areas with clear signs of chemosynthetic life, presumably taking advantage of the increased local productivity^16,27–30^. Although limited, previous research has hinted at the nutritional links between CWC and chemosynthetic bacteria in reducing environments. For example, stable carbon-isotope values previously reported from corals inhabiting the Florida Escarpment seeps were indicative of methane-derived carbon^31^. More recently, studies have successfully identified associations and possible nutritional links between species in the Gulf of Mexico and thiotrophic bacteria within the SUP05 clade, known to be symbionts of various other invertebrates^8,32^. However, a large gap in our knowledge exists regarding the ecology of these seep corals, and the relationships they form with chemosynthetic microbes.

Here, by employing an interdisciplinary approach, combining broad-scale seafloor surveys and habitat suitability modelling with isotope analysis, labelling experiments, and microbial metabarcoding, we investigate the use of seep habitats by a recently discovered cold-water coral, *Swiftia sahlingi*^28^*.* Our findings suggest that this species exploits a specific ecological niche within seep habitats, settling on hard substrates in closer proximity to active seepage than typically observed for cold-water corals. This positioning may facilitate nutritional associations with thiotrophic and potentially methanotrophic bacteria, contributing to the species’ success in an otherwise food-limited environment.

## Materials and methods

### Sampling

**Fig. 1:**
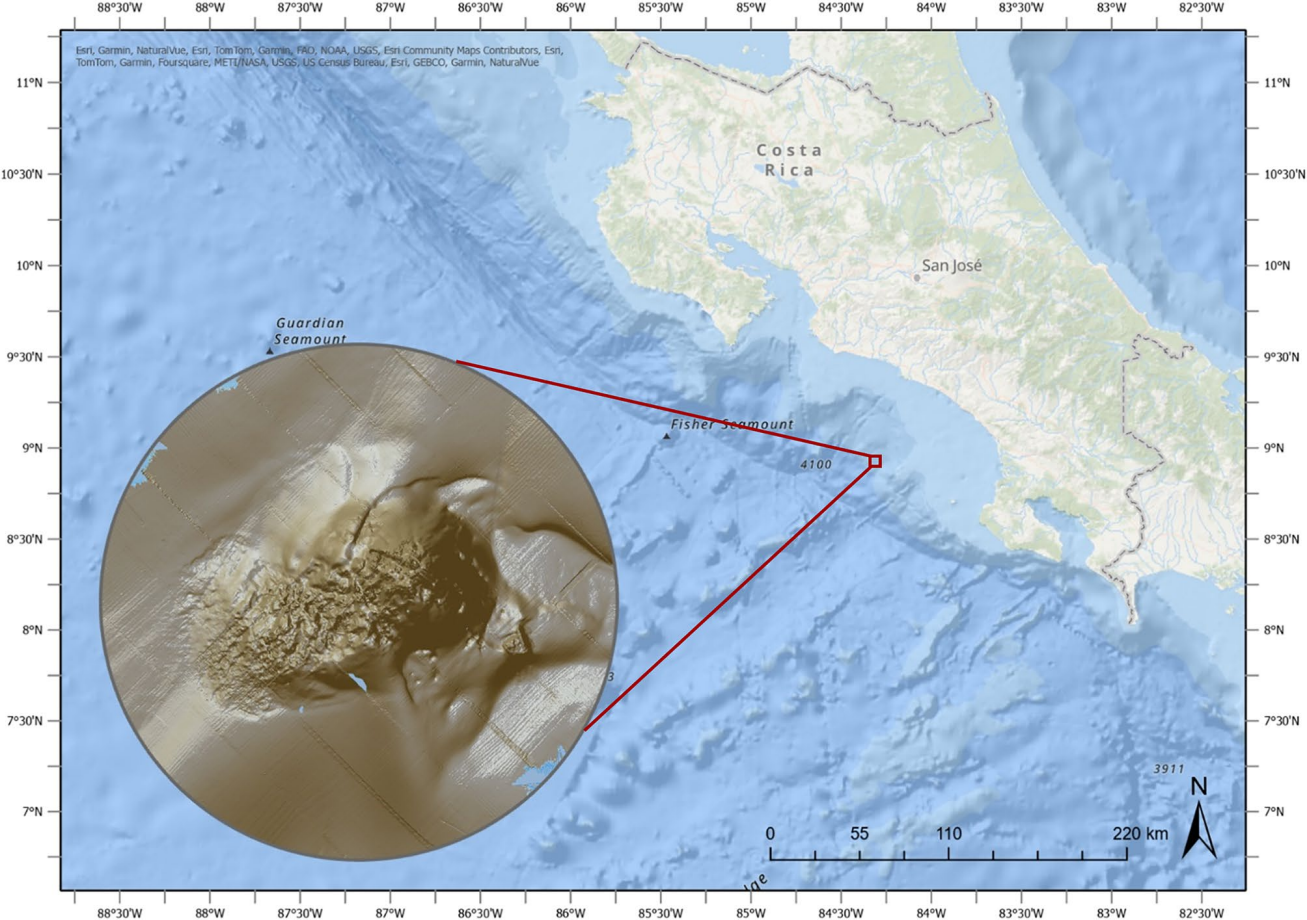
Location and bathymetry of Mound 12, a known methane seep site found on the Costa Rican Pacific continental margin at ~ 1000 m depth.

All *S. sahlingi* samples were collected from Mound 12, a carbonate mound with active methane- and sulfide-rich seepage^33^, located at ~ 1000 m depth on the Eastern Pacific Costa Rican margin (Fig. 1). This site, characterized by ongoing fluid flux and extensive chemosynthetic communities, represents a long-lived seep ecosystem, with carbonate accumulation and geochemical evidence indicating sustained seepage over thousands of years^34–36^.

Samples were obtained from both active and transition areas using the HOV *Alvin* during R/V *Atlantis* expedition AT37-13 (May to June 2017) and with ROV *SuBastian* during R/V *Falkor* expedition FK190106 (January 2019). Corals were maintained throughout the collection process at low temperatures (4 – 10 °C) and subsampled immediately after each dive for various analyses and/or shipboard incubation experiments. Representative species from the coral genera *Acanthogorgia*, *Anthomastus*, *Bathypathes*, and *Callogorgia* were also collected from Mound 12 and neighboring seep sites for isotopic comparison during R/V Atlantis expeditions AT37-13 and AT42-03 (October 2018) using the HOV Alvin.

### Seafloor surveys with AUV Sentry

We used WHOI’s AUV *Sentry*, equipped with sidescan sonar, a Reson 7125 multibeam echosounder, and a downward-looking digital still camera to collect local bathymetric and photographic data. In 2017 and 2018, AUV *Sentry* completed four dives at Mound 12 (dives 431, 432, 502, 507), covering a total distance of 81.66 km. During the photographic portions of each dive, AUV *Sentry* followed a lawnmower survey pattern along a preset navigation path at an average speed of 0.57 m/s, taking approximately 20 photos per minute at an altitude of ~ 5 m. A total of 37,724 photos were collected, of which 150 were annotated with *S. sahlingi* presence. Images with poor visibility (< 2%) were excluded from the analysis.

For each analyzed photo, the substrate and biogenic habitat type were recorded. Substrate type included soft sediment, carbonate rock and shell hash, while biogenic habitats included obligate seep foundation species such as vestimentiferan tubeworms, bathymodiolin mussels, vesicomyid clams and bacterial mats. Given that the occurrence of these obligate seep species depends on access to reduced chemical compounds, areas containing at least one seep indicator species were used as proxies for active seepage. In contrast, areas where carbonate rock was present but seep indicator species were absent were categorized as transition zones.

In ArcGIS Pro, presence points of *S. sahlingi* were used to map its distribution across Mound 12 in relation to seep foundation species and carbonate rock (Fig. S1). A 25 m^2^ (5 m × 5 m) spatial grid was created using the Fishnet tool to partition the study site into spatially explicit cells. Each cell was classified as either “active” or “transition” based on the presence or absence of biogenic seep indicator species. Corals were classified as seep-associated when located within a grid cell containing at least one seep indicator species, and as transition-associated when found only with exposed carbonate rock. To assess spatial relationships with seepage, distances from each *S. sahlingi* presence point to the nearest seep indicator species were calculated using the *Near* tool.

### Habitat suitability models

Correlative habitat suitability models (HSMs), also known as species distribution or ecological niche models, were used to assess the drivers of *S. sahlingi* occurrence throughout Mound 12. The initial variable set used to fit HSMs included bathymetry-derived terrain (hereafter terrain) variables along with Oxidative Reductive Potential (ORP) and temperature from sensors mounted on AUV *Sentry*. Terrain variables were generated from the gridded bathymetric data (1 m resolution) from AUV *Sentry* using the Benthic Terrain Modeler extension in ArcGIS^37^. These included Bathymetric Position Indices (BPI), which measure the height of a given grid cell relative to a surrounding neighborhood. We generated BPI layers at 10 m and 100 m scales. In addition, we generated slope, northness (cosine of aspect), eastness (sine of aspect), and vector rugosity (with a 25 m neighborhood), and curvature layers. These terrain indices have all been used extensively in predictive modeling studies of cold-water corals as proxies for interactions between benthic currents and topography (see ^38^ for extended discussion of these terrain indices).

In addition, we mapped the distance of the center of each grid cell to locations of three biogenic cold seep indicators identified from AUV images including bacterial mats, bathymodiolin mussels and vestimentiferan tubeworms, as well as authigenic carbonates. These features represent successive stages of cold seep evolution, with bacterial mats and mussels indicating early to mid-successional stages and ongoing fluid flux at the seafloor, tubeworms reflecting more established seep habitats often associated with reduced surface seepage, and carbonates representing legacy structures from past seepage^39^. Variables with high correlations to one another (Pearson’s r > 0.7) were sequentially removed before fitting HSMs. The distance-to-mussels, distance-to-mats and distance-to-tubeworms variables were included in the HSMs despite (0.73–0.75) Pearson’s r between them (Fig. S3) because they represent different hypothesized stages of cold seep succession^39^, and thus were of interest in elucidating the potential affinity of *S. sahlingi* to active seepage.

HSMs fit with singular methods are more prone to biases and underperform when compared to ensemble models^40^. Thus, we fit HSMs with the tree-based Random Forests (RF) and Gradient Boosting Machines (GBM) algorithms that tend to be among the highest-performing single methods^41^. These approaches were also selected due to their flexibility in fitting variable interactions among predictors while being relatively insensitive to outliers, multicollinearity, and inclusion of extraneous predictors^42,43^. *S. sahlingi* occurrence points were used to train HSMs generated from photo annotation. Duplicate occurrences within each grid cell were removed, giving a total of 155 unique occurrences on the 1 m grid. 5,000 pseudoabsence or ‘background’ points were dispersed throughout the study area.

Models were trained and evaluated with spatial-block cross-validation, in which the sampling region is split into evenly spaced grid cells or ‘blocks’. Each block is then randomly assigned to one of five cross-validation folds, and HSMs are trained with presence/pseudoabsence data from four of the five folds with data in the other being set aside for evaluation. This process is iterated until each successive block has been used to evaluate the HSMs. This method lessens biases associated with the more commonly used random cross-validation, where spatial transferability of HSMs can be limited by spatial autocorrelation in testing data^44^. For our purpose, we used 50 m blocks (Fig. S4), which was selected to limit spatial autocorrelation in the bathymetric data using the sb.autorange() function in the ‘blockCV’ R package^44^. Area under the receiver operating curve (AUC) of the receiver operating characteristic and true skill statistic (TSS) metrics were used to evaluate HSM performance on testing data. Variable importance for each HSM was estimated by randomly permuting the values of each predictor and measuring the corresponding decrease in the correlation between observations and model predictions. If a variable is important to the model, shuffling its values reduces predictive accuracy. Importance scores were computed as 1 minus the correlation between the original predictions and the predictions based on the permuted variable. This process was repeated 99 times for each variable to ensure stability of the importance estimates. The resulting variable importance values are unitless and relative, with higher values indicating greater influence on model predictions. The different model types were then ensembled by averaging across each of the individual model runs to get a final ensemble mean habitat suitability index (HSI), with each model weighted by its cross-validation performance as measured by TSS. All HSM fitting and evaluation was completed in R^45^ with the ‘biomod2^46^ package.

### Stable isotope analysis

Coral tissue samples were subsampled at sea, rinsed with distilled water, and stored at -20 °C. In the laboratory, all samples were then dried at 60 °C, homogenized into a fine powder and acidified using 10% HCl to remove any inorganic carbon. Tissues were dried again, and 0.5—1 mg of each sample were packaged into tin capsules before being sent to the Laboratory for Isotopes and Metals in the Environment (LIME) at Penn State University for analysis. Stable carbon and nitrogen isotopes were analysed using a Costech ECS 4010 combustion elemental analyzer coupled to a Thermo Delta V isotope ratio mass spectrometer (EA-IRMS) via a ConFlo IV interface. Values are reported in delta notation as per mil (‰) deviations from internationally recognized standard reference materials (Vienna Pee Dee Belemnite (VPDB) for carbon and atmospheric nitrogen (AIR) for nitrogen).

### Shipboard isotope labelling experiments

To assess potential interactions between *S. sahlingi* corals and methanotrophic bacteria, short-term stable isotope incubation experiments with ^13^C-labeled methane (^13^CH₄) were conducted at sea during cruise FK190106. Six *S. sahlingi* colonies (~ 4 cm in size) were collected from Mound 12: three from active seep areas (adjacent to chemosymbiotic fauna including tubeworms and mussels) and three from transition zones, which served as controls due to the lack of visible seep activity or associated symbiotic fauna.

Upon recovery, subsamples were taken from all colonies before incubation at 4 °C for one week in sealed Mylar bags containing 15 mL of 0.2-µm filtered bottom seawater. At the beginning of the incubation, 5 mL of ^13^CH₄ was added to each bag, representing approximately one-quarter of the total headspace. Following the incubation period, each coral was rinsed in distilled water, frozen at -20 °C, and processed for stable isotope analysis as described in the isotope analysis section above.

### Microbial analysis

To assess the broader diversity and potential specificity of microbial associations with *S. sahlingi* in seep habitats, we sequenced six coral colonies from active seep areas at Mound 12. Three of these were the same active specimens used in the incubation experiment, while the remaining three were from other active areas of Mound 12 to evaluate variability in microbial communities across the site. Total genomic DNA was extracted from polyp tissue (~ 50 mg) using the Qiagen DNeasy Blood & Tissue Kit (Qiagen, Valencia, CA, USA) according to the manufacturer’s protocol. The V4-V5 region of the 16S rRNA gene was amplified using bacterial primers with Illumina (San Diego, CA, USA) adapters on the 5′ end 515F (5′-TCGTCGGCAGCGTCAGATGTGTATAAGAGACAGGTGCCAGCMGCCGCGGTAA-3′) and 806R (5′-GTCTCGTGGGCTCGGAGATGTGTATAAGAGACAGGGACTACHVGGGTWTCTAAT-3′;^47^. The PCR reaction mix was set up in duplicate for each sample with Q5 Hot Start High-Fidelity 2 × Master Mix (New England Biolabs, Ipswich, MA, USA) and annealing conditions of 54 °C for 25 cycles. Duplicate PCR samples were then pooled, and 2.5 μl of each product was barcoded with Illumina NexteraXT index 2 Primers that include unique 8-bp barcodes (P5 5′-AATGATACGGCGACCACCGAGATC-TACAC-XXXXXXXX-TCGTCG GCAGCGTC-3′ and P7 5′-CAAGCAGAAGACGGCAT-ACGAGAT-XXXXXXXX-GTCTCGTGGGCTCGG-3′). Secondary amplification with barcoded primers used conditions of 66 °C annealing temperature and 10 cycles. Products were purified using Millipore-Sigma (St. Louis, MO, USA) MultiScreen Plate MSNU03010 with a vacuum manifold and quantified using Thermo Fisher Scientific (Waltham, MA, USA) QuantIT PicoGreen dsDNA Assay Kit P11496 on the BioRad CFX96 Touch Real-Time PCR Detection System. Barcoded samples were combined in equimolar amounts into a single tube and purified with Qiagen PCR Purification Kit 28,104 before submission to Laragen (Culver City, CA, USA) for 2 × 250 bp paired end analysis on the Illumina MiSeq platform with PhiX addition of 20%.

Amplicon sequence data were processed using Quantitative Insights Into Microbial Ecology (QIIME v1.8.0;^48^). Raw sequence pairs were joined and quality-trimmed using default QIIME parameters. Sequences were then clustered at 99% similarity using the UCLUST open reference clustering protocol, and the most abundant sequence in each cluster was selected as the representative. Taxonomic identification for each representative sequence was assigned using the SILVA-138 database and verified via BLAST (www.ncbi.nlm.nih.gov). Representative 16S rRNA sequences were compared to publicly available sequences using BLAST against the NCBI database to assess sequence similarity. The raw Illumina 16S rRNA barcode sequences and associated metadata from this study are available in the NCBI Sequence Read Archive (BioProject #PRJNA1085978).

## Results

**Fig. 2:**
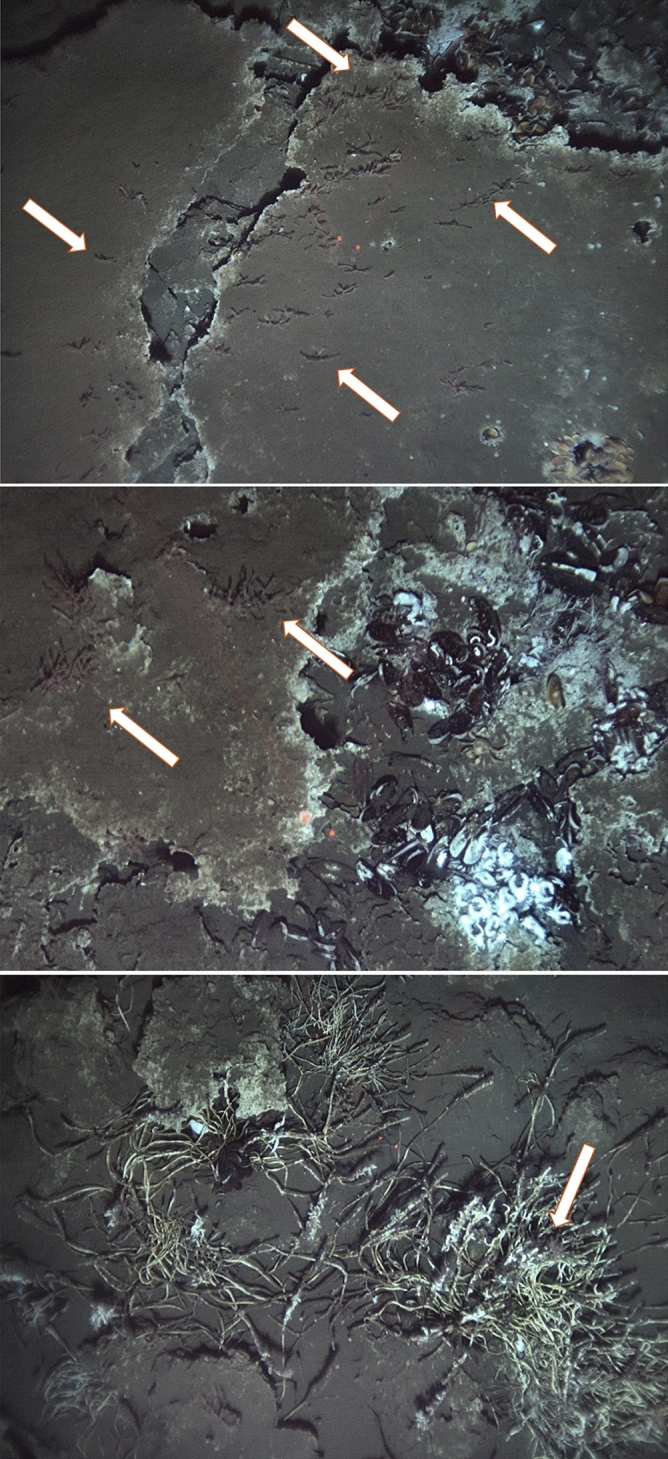
*S. sahlingi* on (A) large carbonate pavements next to bathymodiolin mussels, (B) bathymodiolin mussels and yeti crabs and (C) on vestimentiferan tubeworms. Arrows indicate the presence of corals.

Using the AUV *Sentry*, the distribution of *S. sahlingi* was mapped across the Mound 12 region. Overall, *S. sahlingi* was found to occur over at least 3125 m^2^ at Mound 12 and was, on average, approximately 4 m (± 6 m SD) from active seepage locations, with many occurring within 1 m and in some cases, directly attached to chemosymbiotic tubeworms or mussels (Fig. 2).

**Fig. 3:**
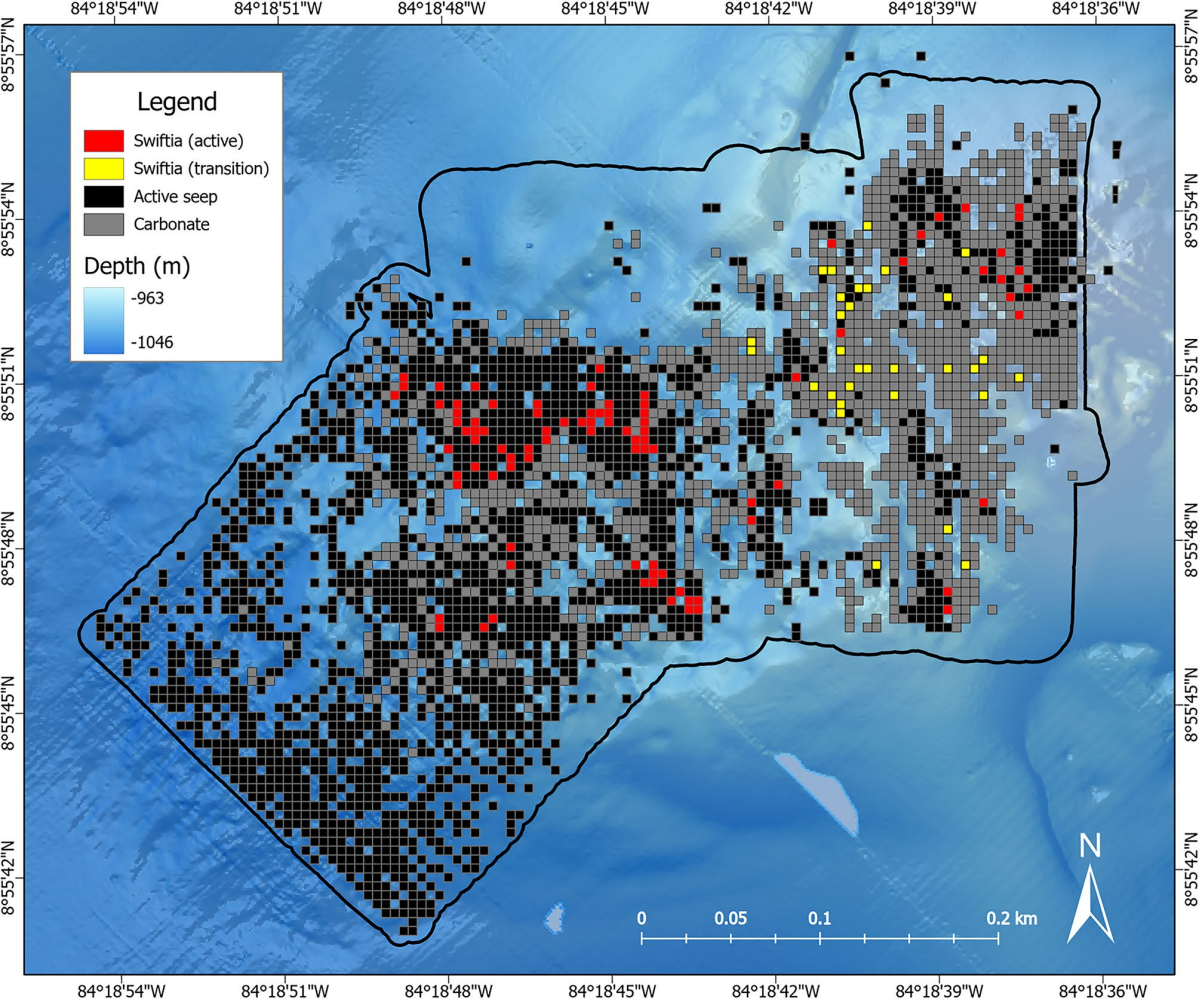
Distributions of seep (active) and non-seep (transition) associated *S. sahlingi* across Mound 12. Areas with *S. sahlingi* associated with chemosymbiotic seep indicator species are show in red, while *S. sahlingi* found in transition zones are shown in yellow. Active seep areas without corals are represented by black grid cells, while transition areas without corals are represented by grey cells.

Notably, mapping results revealed that the majority of *S. sahlingi* observations occurred in active seep zones (79%, n = 119; Fig. 3). The remaining 21% (n = 31) were observed in transition zones characterized by abundant carbonate rock, at an average distance of 12 m (± 9 m SD) from active seepage.

### Distribution and habitat preferences

**Fig. 4:**
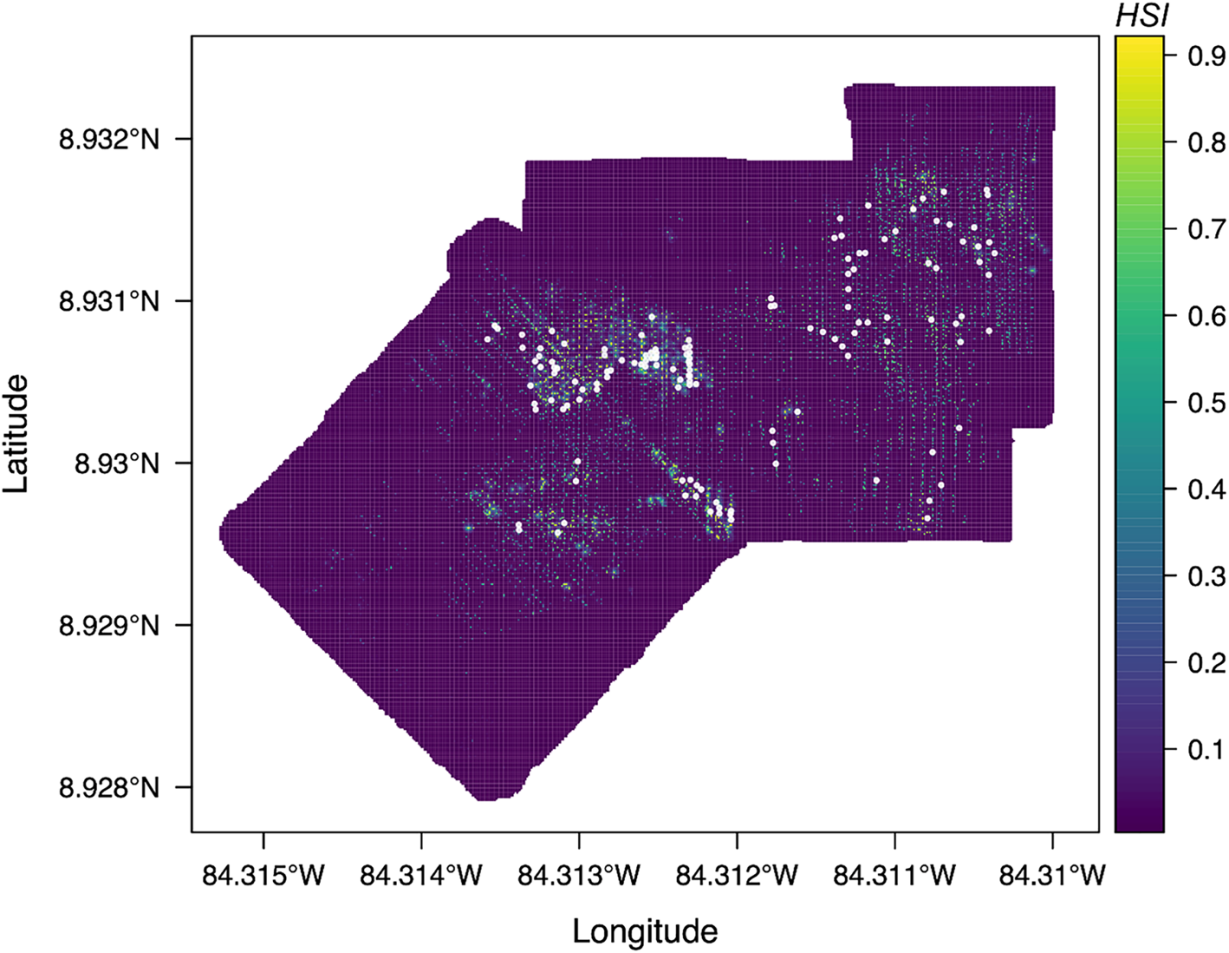
Ensemble weighted mean habitat suitability index (HSI) scores for *S. sahlingi* at Mound 12 with *S. sahlingi* occurrences overlain (white points). Note that HSI scores are on a 0–1 scale.

HSMs predicting *S. sahlingi* distribution throughout Mound 12 had high AUC (> 0.92) and TSS (> 0.8) scores for all models (Fig. S5). Both algorithms performed similarly , with GBM models slightly outperforming the RF models, including individual runs with TSS > 0.9 (Fig. S5). Habitat suitability index (HSI) scores predicted throughout the study area show high fidelity to the observed distribution of *S. sahlingi* (Fig. 4).

**Fig. 5:**
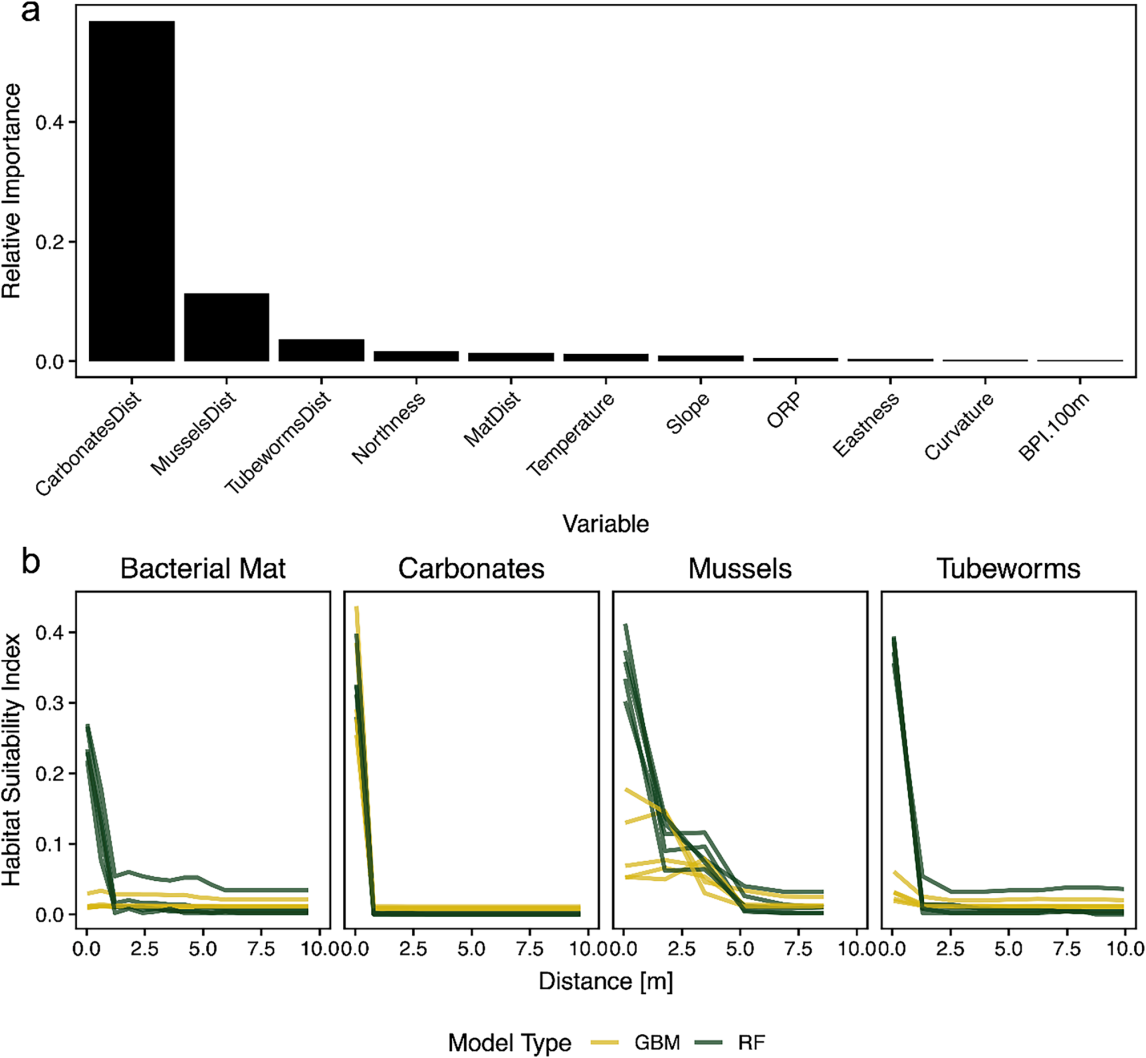
Distance to authigenic carbonates and biogenic seep indicators are the primary site-scale drivers of *S. sahlingi*. Relative variable importance (a) and response curves (b) illustrating the partial effects of distance-to-seep indicator predictors on Habitat Suitability Index from HSMs fit with different algorithms (colors). See Figure S6-S7 for all response curves from HSMs.

Carbonates and seep indicator species were the overwhelming drivers of *S. sahlingi* distribution. The only variables with mean relative importance scores across all models above 0.1 were the distance-to-carbonate (0.57) and distance-to-mussels (0.11) variables, with the former being the most important by a wide margin (Fig. 5A). All other variables had relatively little effect on the HSMs. Response curves across the range predictor variables show that for all seep indicators, the HSI increases sharply within ~ 10 m of the indicator (Fig. 5B). The pattern of increased habitat suitability within ~ 2 m of authigenic carbonates was the only pattern found consistently in all model runs and with the same shape and effect between algorithms (Fig. 5B), indicating it was consistent across the areas of Mound 12 as each model run was based on different spatial subsections of the data (Fig. S4).

### Isotopic evidence of chemosynthetic nutritional input

**Fig. 6:**
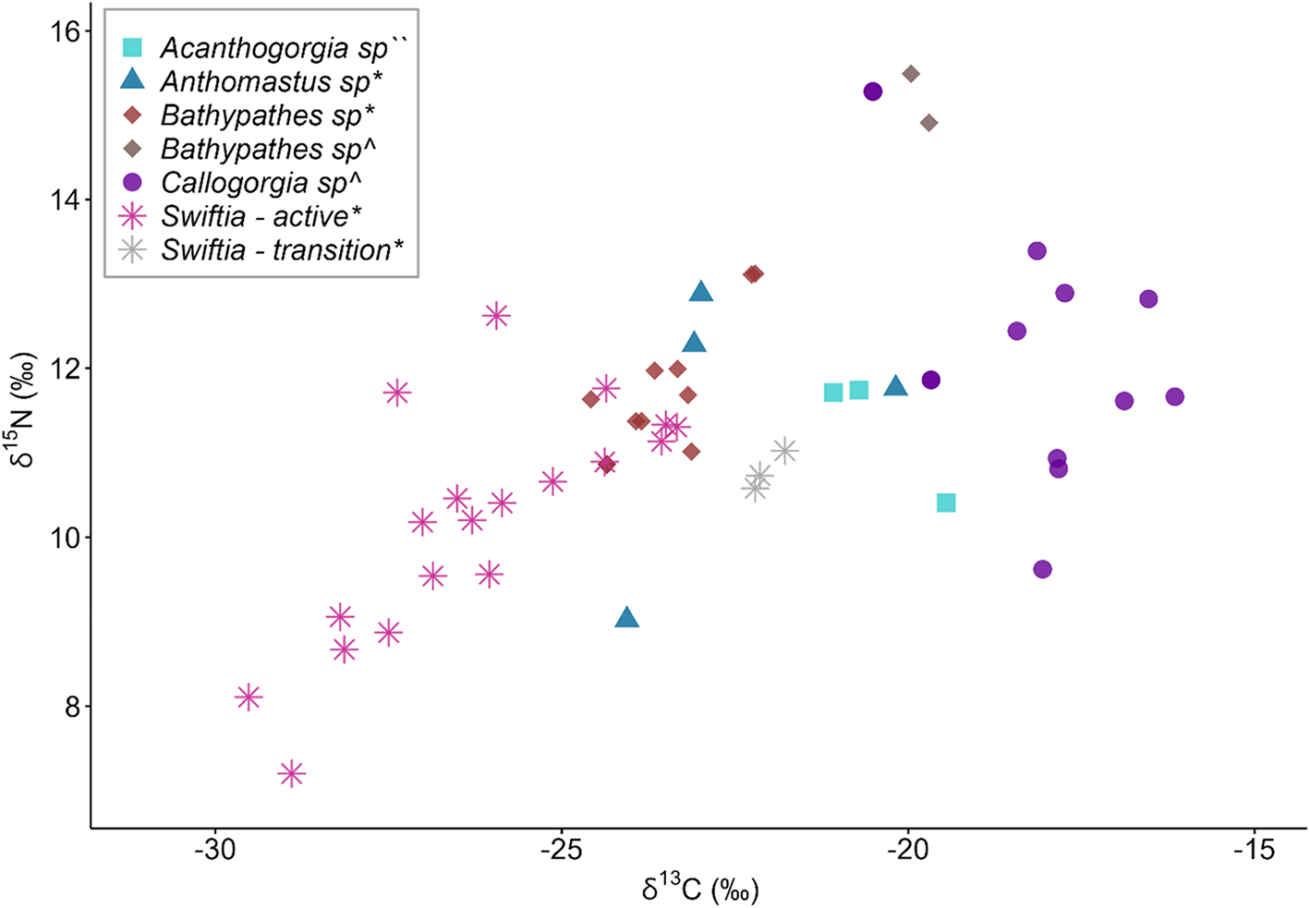
Stable isotope biplots (δ^13^C vs. δ^15^N) of *S. sahlingi* and other corals from methane seep areas along the Pacific Costa Rica margin: Mound 12 (*), Jaco Scar (^), and Quepos Seep (``). Seep-associated *S. sahlingi* are shown as light pink stars, while non-seep-associated individuals collected from transition zones are represented by grey stars.

Tissue stable carbon and nitrogen isotopes of *S. sahlingi* were significantly different from other corals collected from Mound 12 and neighbouring seep sites (ANOVA, F = 33.86, p   < 0.001, Fig. 6). The mean tissue δ^13^C values of *S. sahlingi* were -25.6 ± 2.2‰ (-29.5 to -21.7‰, N = 22; ± 1 SD), even when including more enriched values reported from specimens collected in transition zones. Mean tissue δ^13^C values from other coral species collected from Mound 12 and neighboring seeps were -20.8 ± 2.6‰ (-24.5 to -16.1‰, N = 33; ± 1 SD). Similarly, the tissue δ^15^N values of *S. sahlingi* were significantly more depleted than the surrounding corals (ANOVA, F = 4.76, p = 0.0013, Fig. 6), with mean values of 10.2 ± 1.2‰ (7.2 to 12.6‰, N = 22; ± 1 SD), compared to other species with values of 12.1 ± 1.5‰ (9 to 15.4‰, N = 33; ± 1 SD).

To determine the capability of *S. sahlingi* to assimilate carbon from oxidized methane into its tissues, short-term incubations with ^13^C-labelled methane were set up with live specimens from both active and transition zones. Pre-treatment tissue δ^13^C values of the specimens collected from active areas were significantly more negative than specimens collected from transition zones (Welch’s t-test; t = -9.475, df = 19.981, p  < 0.001, N = 22). Tissue δ^13^C and δ^15^N of *S. sahlingi* from active areas were -26.2 ± 1.8‰ (-29.5 to -23.3‰), and 10.1 ± 1.3‰ (8.1 to 11.3, N = 19, ± 1 SD), respectively. In comparison, tissue δ^13^C and δ^15^N of *S. sahlingi* from transition zones were -22 ± 0.2‰ (-22.2 to -21.7‰), and 10.7 ± 0.2‰ (10.5 to 11, N = 3, ± 1 SD).

After seven days of incubation, specimens from transition zones showed no enrichment in δ^13^C values (–24.8 to –23.5‰; N = 3, ± 0.6‰), consistent with pre-treatment levels. Specimens from actively seeping areas, however, showed a more variable response. While most exhibited little change, one individual displayed a substantial isotopic enrichment, with tissue δ^13^C increasing from –28.1 to + 34.1‰ (Table 1). This pronounced shift in carbon isotopic composition indicates significant uptake of ^13^C from the incubation environment, consistent with potential assimilation of labelled methane-derived carbon.

**Table 1:** Stable carbon (δ^13^C) and nitrogen (δ^15^N) isotope values of S. sahlingi collected from dive SO215, measured prior to (Native) and following (Incubated) incubation with ^13^C-labelled methane. Samples denoted D–F correspond to the same individuals used in microbial metabarcoding analyses (see Fig. 7 & S7).

**Dive/Sample**	**Habitat**	**Native**		**Incubated**	
		**δ**^**13**^**C**	**δ**^**15**^**N**	**δ**^**13**^**C**	**δ**^**15**^**N**
SO215 (D)	Active seep	-24.86	9.54	-26.84	10.37
SO215 (E)	Active seep	-28.9	7.2	-27.35	7.91
SO215 (F)	Active seep	-28.14	8.67	34.1	10.2
SO215	Transition	-22.14	10.73	-23.59	10.29
SO215	Transition	-22.21	10.58	-24.86	10.72
SO215	Transition	-21.78	11.02	-23.74	10.66

**Fig. 7:**
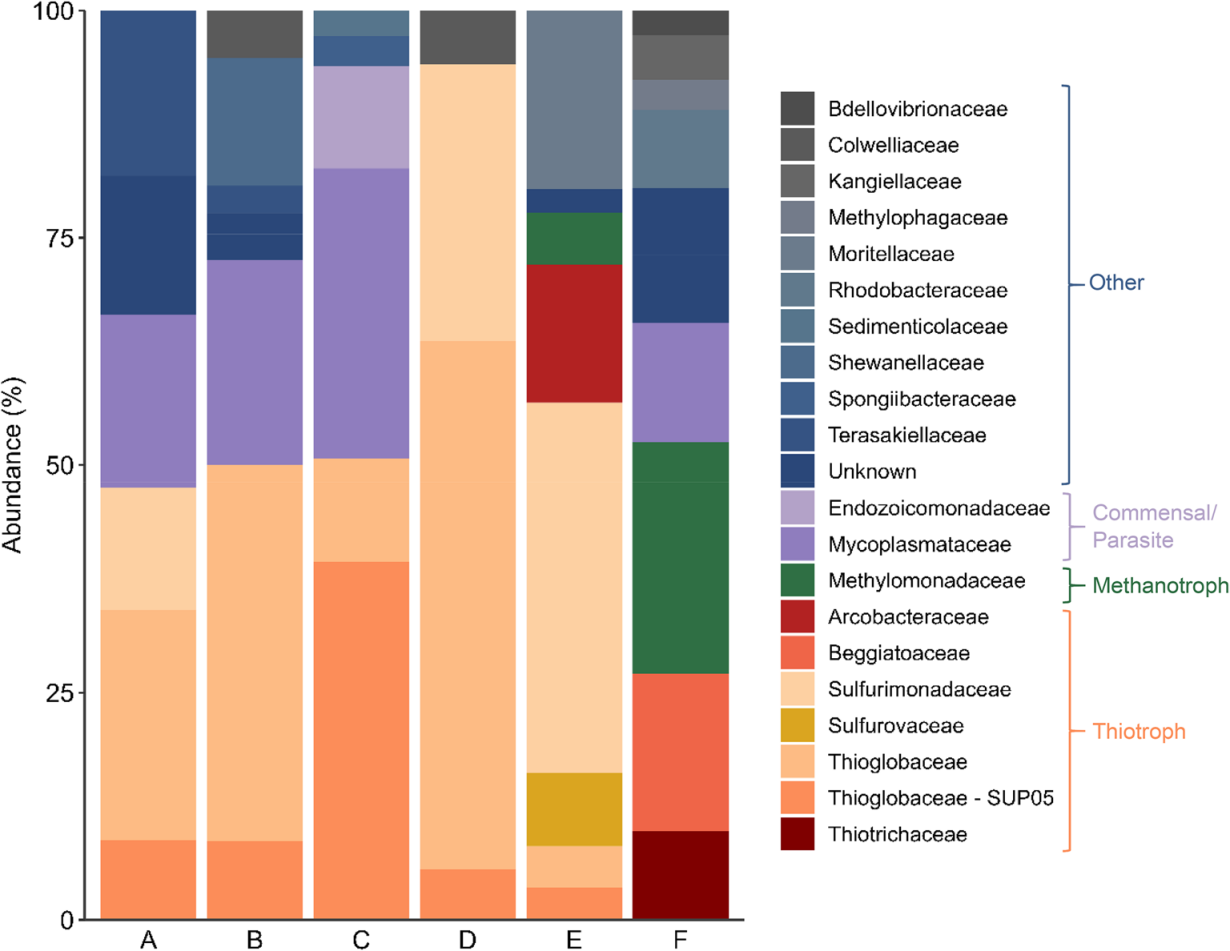
Bacterial community structure for *Swiftia sahlingi* collected from active areas of Mound 12. Phylotypes are grouped to 99% 16S rRNA sequence similarity*.* Samples D-F correspond to those used in the ^13^CH₄ incubation experiment (See Table. 1 & Fig. S9); sample F exhibited evidence of ^13^C incorporation. Samples A-C represent additional colonies from active areas included to assess spatial variability in microbial community composition.

The variation in the δ^13^C values observed in the specimens from active areas post-incubation is likely driven by differences in their associated microbial communities. Methane-oxidizing bacteria were detected in only two of the specimens used in the incubation experiment, where their relative abundance ranged from 5 to 20% (Fig. 7). Notably, the specimen that exhibited a significant uptake of ^13^CH_4_ also had the highest relative abundance of methane-oxidizing bacteria (Fig. 7).

### Molecular evidence for chemosynthetic microbial associations

Bacterial community analysis of *S. sahlingi* via 16S rRNA Illumina barcoding revealed a composition dominated by sulfur-oxidizing families, comprising 24—91% of the total bacterial community (N = 6; Fig. 7). Among these, the SUP05 clade of Gammaproteobacteria was present in most samples (3—35%, N = 5; Fig. 7), along with other taxa within the Thioglobaceae and Sulfurimonadaceae families (Fig. 7), many of which have been reported from deep-sea chemosynthetic environments^7,32^, including as putative symbionts of various deep-sea invertebrates^49–51^. We identified 26 distinct SUP05 phylotypes, but only three were prevalent, each representing more than 10% of the SUP05 reads in at least one sample.

While thiotrophic groups dominated the total bacterial communities, methanotrophic bacteria within the Methylomonadaceae family were also identified in two coral specimens (Fig. 7; samples E–F). Specifically, members of the Methylococcales Marine Methylotrophic Group 2 (MMG-2) comprised 5–20% of the total bacterial community in these samples. Within MMG-2, 28 distinct phylotypes were observed, though only three phylotypes were dominant, each exceeding 10% of MMG-2 reads in at least one sample.

## Discussion

Deep-sea methane seeps are remarkable habitats where novel adaptations often arise among inhabitants in response to unique environmental conditions. While some studies have reported the presence of cold-water corals (CWC) in and around chemosynthetic habitats, their ecology has largely been overlooked, with the prevailing assumption that these corals simply exploit the hard authigenic carbonate substrates left behind once seepage has subsided. Until recently, there has been little evidence demonstrating the potential benefits that corals may gain by inhabiting chemosynthetic environments^32,32^. Here, we show that seep-derived productivity is important to a recently discovered seep-dwelling octocoral and provide evidence that this species likely incorporates seep-derived carbon as a nutritional source.

Since their discovery, it has generally been assumed that cold-water octocorals acquire most of their nutrients from sinking POM via passive suspension feeding, mainly due to their low densities of stinging nematocysts^52^. However, growing evidence suggests that CWC inhabiting chemosynthetic environments may form symbiotic associations with chemosynthetic bacteria to meet part of their nutritional demands^8,32^. *Swiftia sahlingi*, a recently discovered octocoral species^28^, is known to inhabit an active methane seep site, Mound 12, located on the Costa Rican Pacific continental margin^53^. Contrary to the common assumption that corals occupy only the periphery of chemosynthetic habitats, this coral has been repeatedly observed thriving in actively seeping areas, often living on or adjacent to endemic foundation species^28^.

Using AUV *Sentry,* we mapped the regional distribution of *S. sahlingi* at methane seep sites along the Pacific Costa Rican margin. Results revealed that *S. sahlingi* occurs in both active and transition areas of the Mound 12 seep, but shows a clear preference for active areas. This was evidenced by frequent co-occurrence with well-known seep-associated fauna and a relative absence from areas characterized solely by carbonate rock with no apparent signs of active seepage. Notably, *S. sahlingi* was not only found living in close proximity to these endemic chemosymbiotic species^1,54,55^, such as bathymodiolin mussels and vestimentiferan tubeworms, but in some cases was observed growing epizootically on these species (Fig. 2).

Habitat suitability models (HSMs) supported the idea that *S. sahlingi* preferentially inhabits areas of active fluid flux at this site (Fig. 4). As predicted, carbonate presence was identified as the strongest driver of *S. sahlingi* distribution, as corals require hard substrata for attachment (Fig. 5). However, distance to mussels also emerged as a significant predictor of suitable habitat, notably even more so than distance to tubeworms. Previous research has shown that methane seeps undergo several successional stages following the onset of fluid flux. For example, the first fauna to colonize these areas are often bathymodiolin mussels^39^, which require access to reduced chemicals near the sediment–water interface^56^. The importance of mussel presence and the steep increase in *S. sahlingi* HSI with proximity to mussels suggest that exposure to fluid flux may likewise support coral persistence. Furthermore, although HSMs did not identify other seep indicators as significant predictors of suitable coral habitat—likely due in part to high collinearity between these variables—response curves showed a sharp increase in *S. sahlingi* occurrence near tubeworms and bacterial mats, strongly suggesting these corals are benefiting from active seepage, albeit with weaker associations than those observed with carbonates.

Stable isotope analysis is an important tool for assessing reduced chemical utilization in chemosynthetic ecosystems, as carbon derived from methane oxidation typically produces very negative δ^13^C values, while sulfide oxidation coupled to carbon fixation can also yield comparably low δ^13^C values^57,58^. Samples of the scleractinian coral *Lophelia pertusa* collected near seep habitats showed isotopic evidence consistent with a diet primarily reliant on phytodetritus, leading to the conclusion that this species likely benefits from increased carbonate availability only after seepage activity has subsided^27,59^. In contrast, previous studies on octocorals have reported carbon and sulfur isotopic signatures consistent with diets composed, at least in part, of seep-derived productivity^29,31^. The relatively light δ^13^C in *S. sahlingi* tissues from active areas (ranging from -29.5 to -23.4‰), compared to other corals from the same area or neighboring seep sites, suggests this species is using a distinct nutritional strategy. These isotopic values are consistent with those previously documented in organisms obtaining carbon fixed from sulfide oxidation via thiotrophic symbionts^60–62^. Therefore, it is plausible that *S. sahlingi* acquires at least some nutrients from chemosynthetically derived carbon, potentially through selective suspension feeding or by adopting a facultative symbiotic relationship with chemosynthetic bacteria.

The values reported here for *S. sahlingi* collected from active areas are similar to those recently reported for corals in the Gulf of Mexico, which have been proposed to be chemosymbiotic^8,32^. Furthermore, δ^13^C values from *S. sahlingi* in active areas are similar to those recently reported in the vent anemone *Ostiactis perseae*, which was found to host thiotrophic endosymbionts^7^. Each of these studies, including the present study, has shown a wide range of tissue δ^13^C values, indicating that the proportion of chemosynthetically derived carbon assimilated into host tissues varies among individuals. This variation suggests that these anthozoans have adopted mixotrophic feeding strategies, combining facultative symbiosis with methanotrophic and thiotrophic bacteria alongside suspension feeding.

The importance of chemosynthetic bacteria to these corals, particularly thiotrophic species, was further evidenced by the bacterial communities associated with each specimen. Sulfur-oxidizing groups dominated the total bacterial communities, comprising up to 91% of the reads in one sample. Notably, SUP05 (Thioglobus) phylotypes were present in most corals examined. These sulfide-oxidizing bacteria are well documented across a variety of marine environments globally^63–66^ and are known to exist either as free-living organisms^67^ or as symbionts of invertebrates such as clams, mussels, and sponges^63,68–70^ at hydrothermal vents, methane seeps, and in oxygen-deficient habitats. This clade was also proposed as a symbiont of corals from methane seeps in the Gulf of Mexico, where they comprised up to 91% of the total bacterial community^8,32^. Bacteria within the Thioglobaceae family were likewise found to be dominant endosymbionts in the anemone, *O. pearseae*, another cnidarian known to inhabit reducing environments^7^. Hosting sulfide-oxidizing bacteria is likely beneficial to these cnidarians in several ways, including nutritional supplementation and detoxification of the surrounding water via oxidation of hydrogen sulfide during respiration.

Interestingly, results also revealed associations with methane-oxidizing bacteria in some of the corals, including a specimen that appeared to incorporate the ^13^C-labelled methane during the incubation period (Fig. 7; sample F). These bacteria, belonging to the MMG-2 group, have been reported from a broad range of marine invertebrate hosts inhabiting methane-rich environments, including several sponge species from the methane seeps in the Gulf of Mexico and mud volcanoes off Barbados^4,71,72^. MMG-2 have also been identified via microscopy and 16S rRNA sequencing in the squat lobster *Shinkaia crosnieri* from hydrothermal vents off Japan^73^, ciliates from the Eastern Pacific^74^, tubeworm species including *Siboglinum poseidoni* in the Gulf of Cadiz^75^ and two fanworm species, *Bispira sp* and *Laminatubus sp,* from Mound 12 and adjacent seeps along the Costa Rica margin^2^. Notably, the MMG-2 phylotypes reported here shared only 97–98% identity with those recovered from these fanworms, and 96% identity with sequences from local water samples, suggesting they may represent distinct strains or potentially different species^2^. In fact, the coral-associated MMG-2 phylotypes shared greater similarity (98–99% identity) with symbionts reported from sponges in the Gulf of Mexico^68^ than with those associated with fanworm species from a nearby seep^2^, suggesting that, while the association appears facultative, it may also reflect a degree of specificity that warrants further investigation. Among the corals incubated with ^13^C-labeled methane, one individual exhibited a marked enrichment in tissue δ^13^C values, indicating potential assimilation of methane-derived carbon. This pattern was not observed across all specimens, suggesting individual variability in either microbial associations or metabolic responses. While thiotrophic bacteria were more prevalent overall, the co-occurrence of methanotrophic groups and the isotopic shift in this specimen support the possibility of facultative associations with methanotrophs. To our knowledge, this is the first evidence suggestive of methane-based carbon assimilation in a deep-sea anthozoan. Although preliminary, these findings warrant further investigation.

The variability in microbial compositions and stable isotope values, together with results from live-animal isotope enrichment incubations and observed distributions across both active and transitional seep areas, support the hypothesis that these anthozoans are capable of adopting mixotrophic feeding strategies. The ability to shift between traditional suspension feeding and forming facultative symbioses with sulfide- and possibly methane-oxidizing bacteria is undoubtedly a beneficial mechanism in highly dynamic chemosynthetic habitats, enabling these corals to extend their ecological niche into reducing environments. While more research is needed to determine the prevalence of these relationships and the exact mechanisms for carbon exchange, our study reveals a previously unrecognized association between CWC and chemosynthetic bacteria, providing new evidence that anthozoans may utilize microbially mediated carbon in reducing environments. These findings also highlight the potential role of methane seeps in supporting cold-water coral ecosystems, which could have important implications for deep-sea conservation and management efforts, particularly in regions targeted for extractive activities.

## Supplementary Information

Supplementary Information.

## Data Availability

16S rRNA barcode sequences and metadata collected in this study are available from the NCBI Small Read Archive (BioProject # PRJNA1085978). All other data are available in the main text or the supplementary materials.
