# Supplementary material for: Microbially mediated carbon utilization by a cold-water coral inhabiting methane seeps
Source: Sci Rep. 2026 Feb 24;16:9603. doi: 10.1038/s41598-025-32153-0 (PMC13009185; doi:10.1038/s41598-025-32153-0)
Supplement: Supplementary file 1 — Supplementary Information. [file 41598_2025_32153_MOESM1_ESM.pdf]

Supplementary figures and tables

Table S1. *Swiftia sahlingi* sample locations, analysis type and dive metadata.

Analysis	Site	Date	Dive
Stable isotope	M12 - Active	May 23, 2017	AD4908
Stable isotope / Microbial metabarcoding	M12 - Active	May 24, 2017	AD4909
Stable isotope / Microbial metabarcoding	M12 - Active	Jun 1, 2017	AD4917
Stable isotope / Microbial metabarcoding	M12 - Active	Jun 5, 2017	AD4922
Stable isotope / Incubation	M12 - Transition	Jan 8, 2019	SO215
Stable isotope / Microbial metabarcoding / Incubation	M12 - Active	Jan 8, 2019	SO215

Table S2. AUV *Sentry* dive information.

Dive	Distance	Speed	Photos	Annotations
431	17.47	0.56	9448	35
432	21.79	0.60	10822	0
502	20.77	0.58	8168	81
507	21.63	0.54	9286	27

Table S3. Stable isotope values of corals collected from Mound 12 and neighboring seep sites along the Pacific Costa Rican margin (mean + SD).

Location	Depth (m)	Coral species	D13C (mean + SD)	D15N (mean + SD)
Quepos seep	400	*Acanthogorgia sp*	-20.4 ± 0.7	11.2 ± 0.6
Mound 12	1000	*Anthomastus sp*	-22.5 ± 1.4	11.4 ± 1.4
Jaco Scar	1600	*Bathypathes sp*	-19.8 ± 0.1	15.2 ± 0.2

Mound 12	1000	*Bathypathes sp*	-23.5 ± 0.7	11.7 ± 0.7
Jaco Scar	1600	*Callogorgia sp*	-18.3 ± 1.3	12.3 ± 1.5
Mound 12	1000	*Swiftia sahlingi*	-25.6 ± 2.1	10.2 ± 1.2

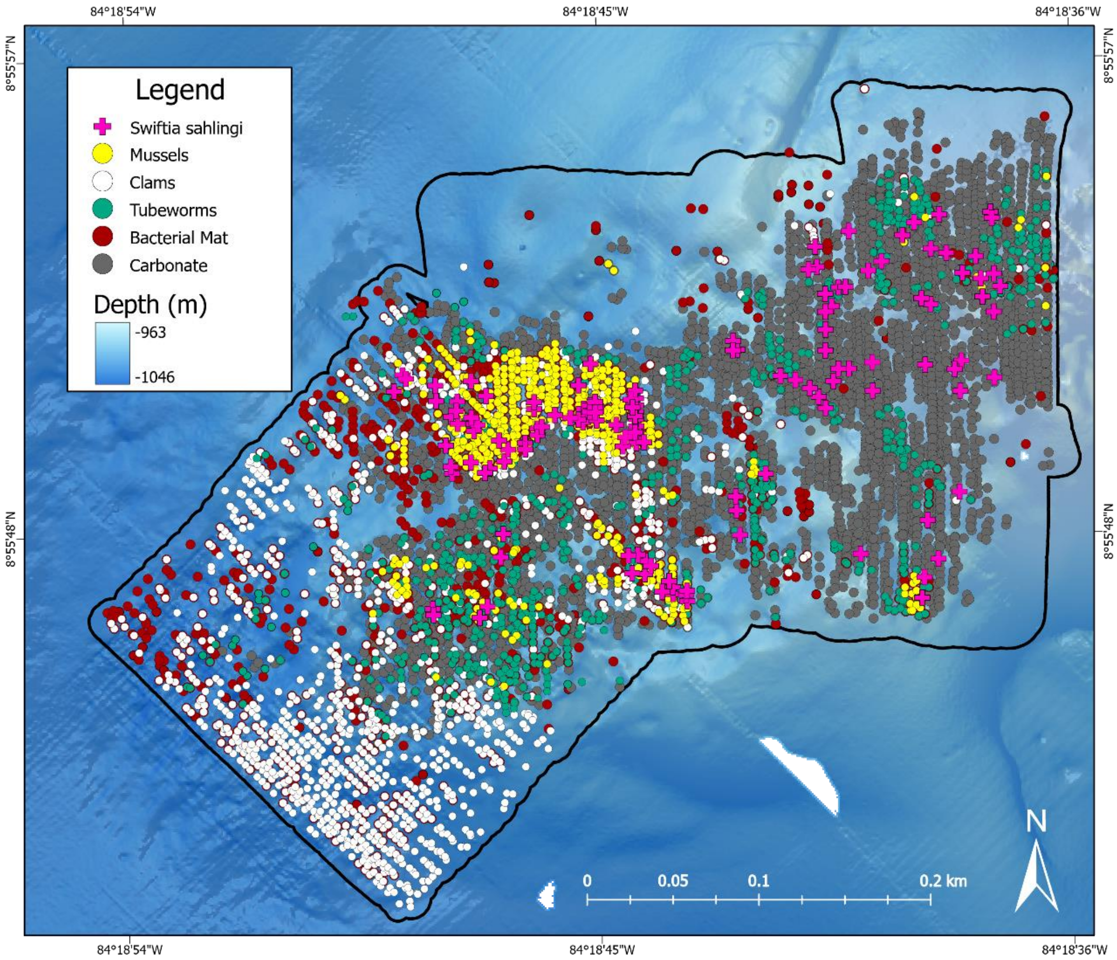

Figure S1. *Distribution of seep-associated fauna and substrates at Mound 12 on the Costa Rican margin (~1000 m depth).* Map shows annotated presence of seep fauna and authigenic carbonates based on raw observational data collected by AUV *Sentry* during seafloor photographic surveys. Data points represent identified occurrences of *Swiftia sahlingi*, mussels, clams, tubeworms, bacterial mats, and carbonate outcrops. Background bathymetry is derived from multibeam sonar data collected during the same survey.

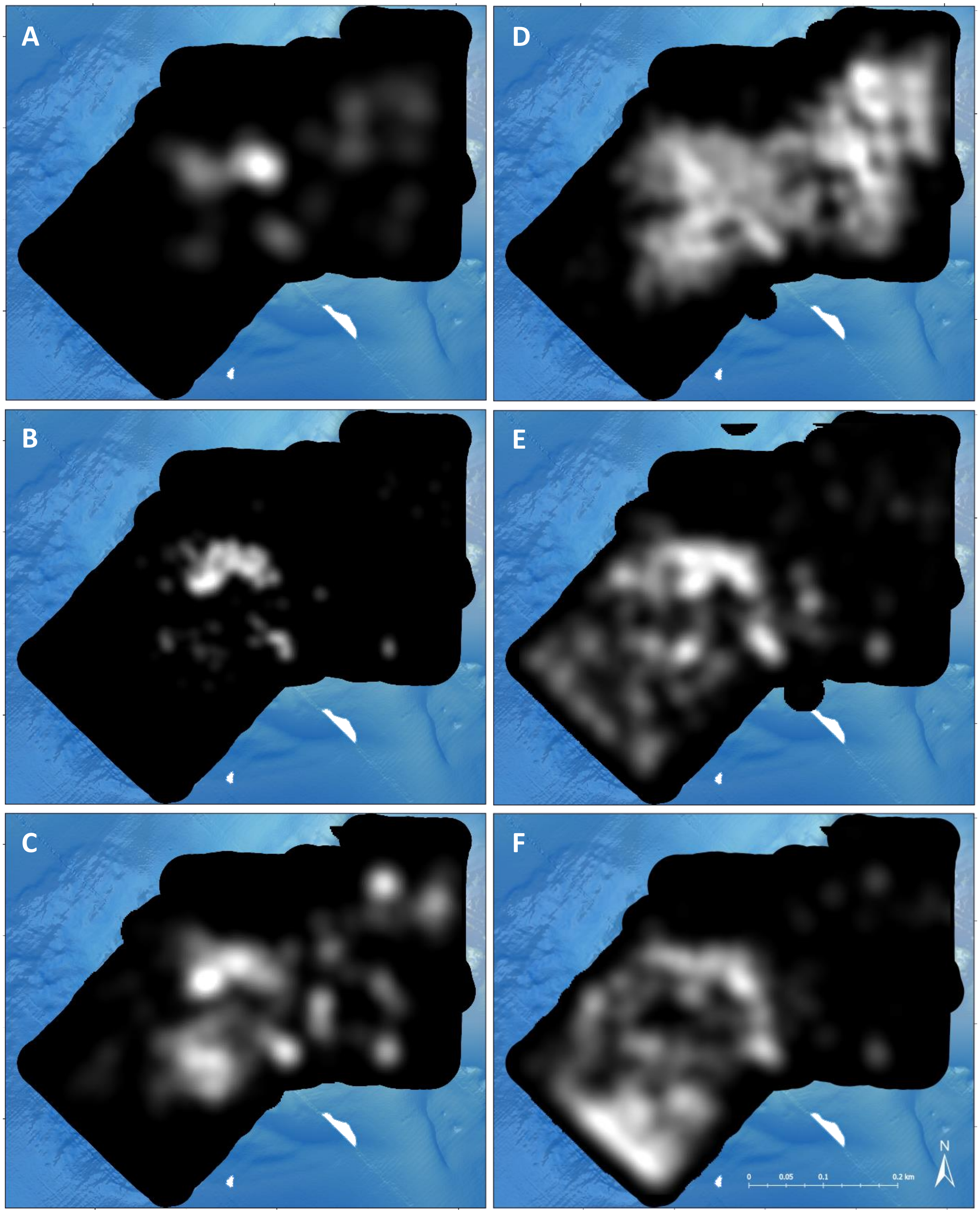

Figure S2. Spatial distribution of seep indicator fauna and carbonate substrate across Mound 12 based on kernel density estimation. Panels show the relative presence of (A) *Swiftia* spp. (corals), (B) mussels, (C) tubeworms, (D) carbonate substrate, (E) bacterial mat, and (F) clams. Density values were calculated using kernel density analysis in ArcGIS Pro. White areas

indicate higher relative presence, with darker shades representing lower or no presence. All panels are shown at the same spatial scale; scale bars represent distance in kilometers.

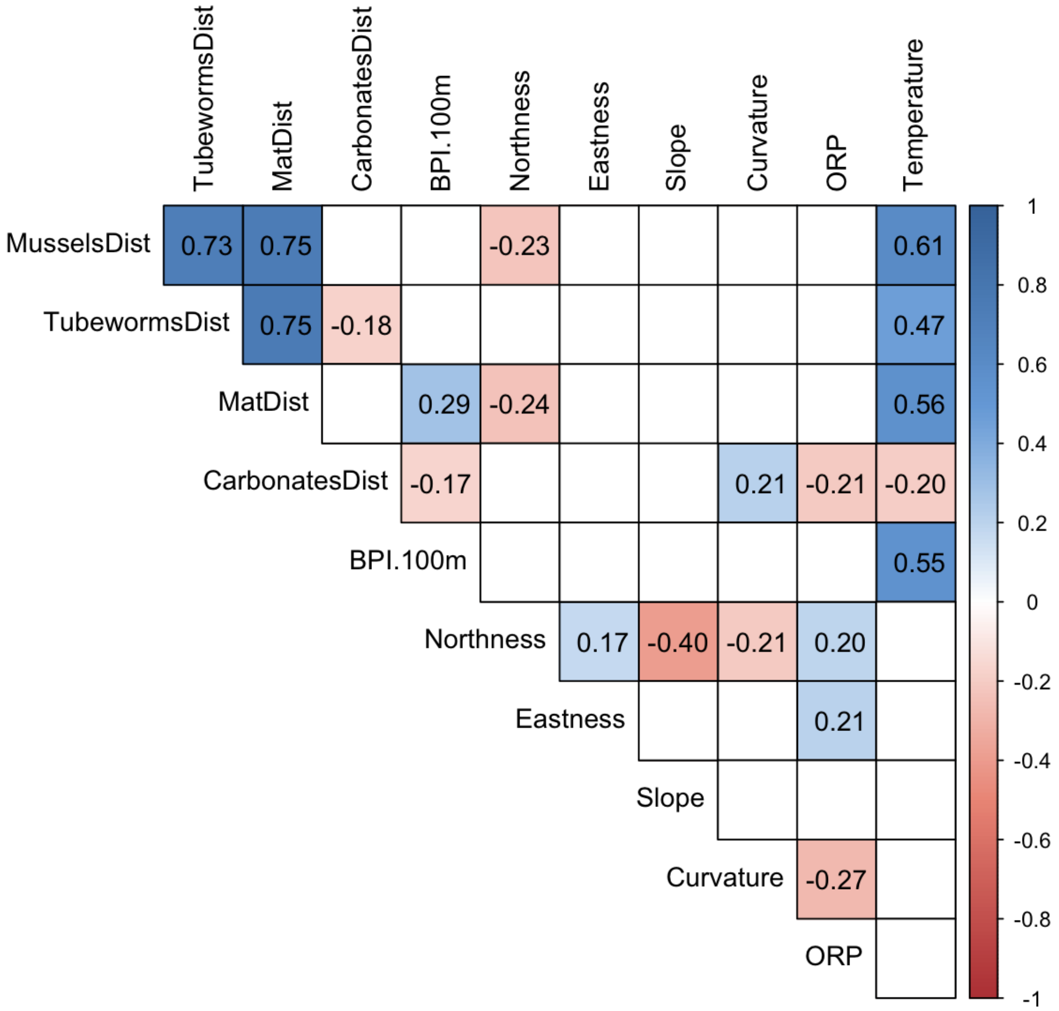

Figure S3. Correlation (Pearson's r) between variables used to train HSMs. Only variables with correlations less than 0.7 were retained.

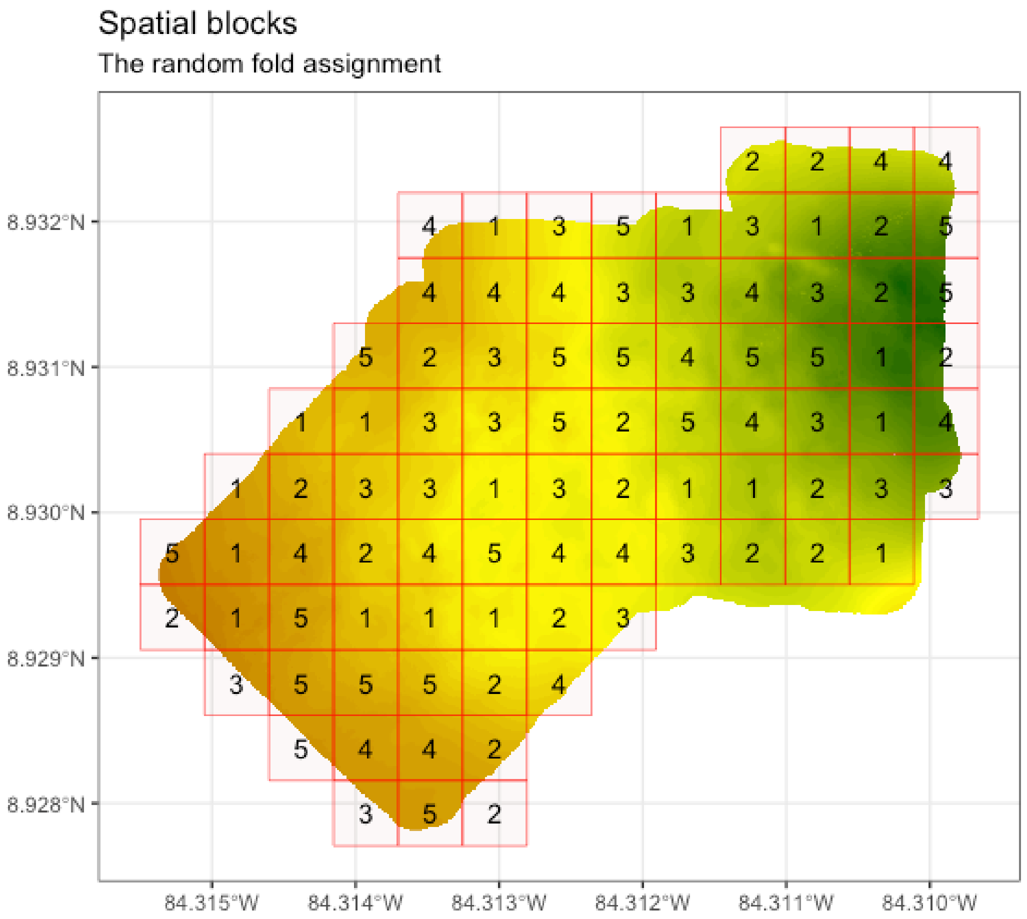

Figure S4. Spatial blocks used for cross-validation of *Swiftia sahlingi* HSMs at Mound 12 (see Methods).

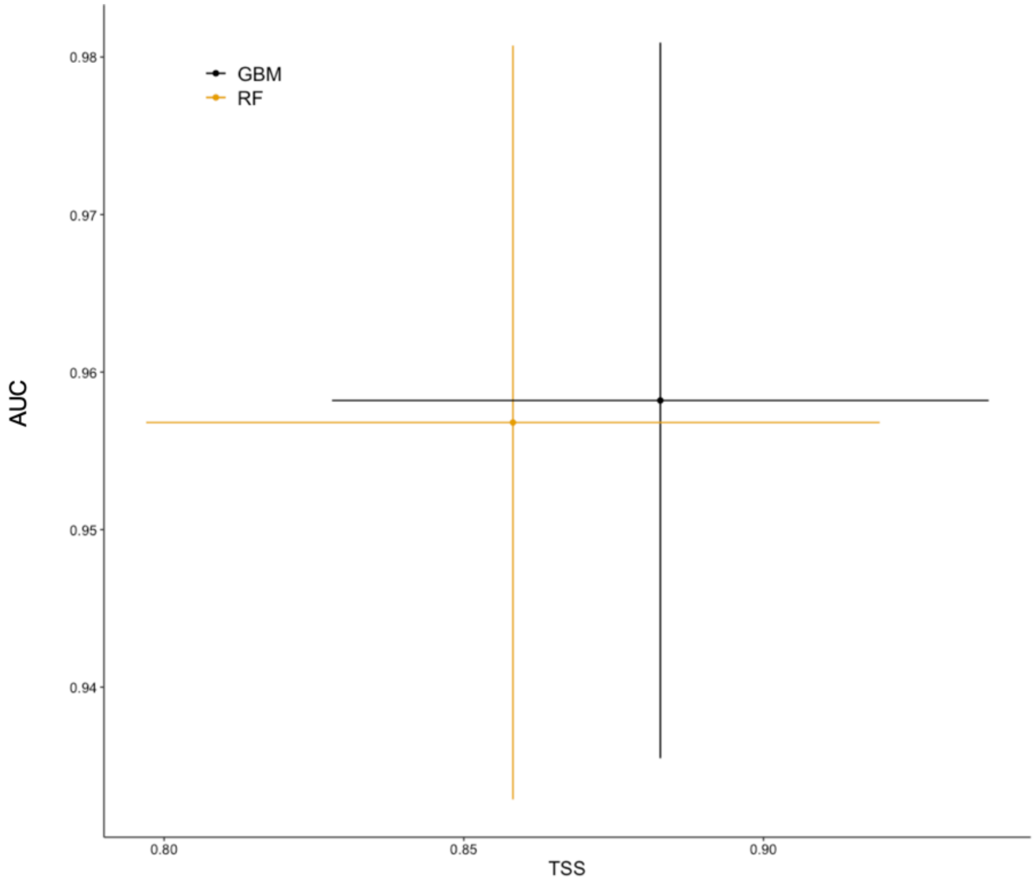

Figure S5. Model evaluation scores from spatial block cross-validation (see Methods). Mean (points) and range (lines) of area under the receiving operator curve (AUC) and true skill statistic (sensitivity + specificity; TSS) scores are shown.

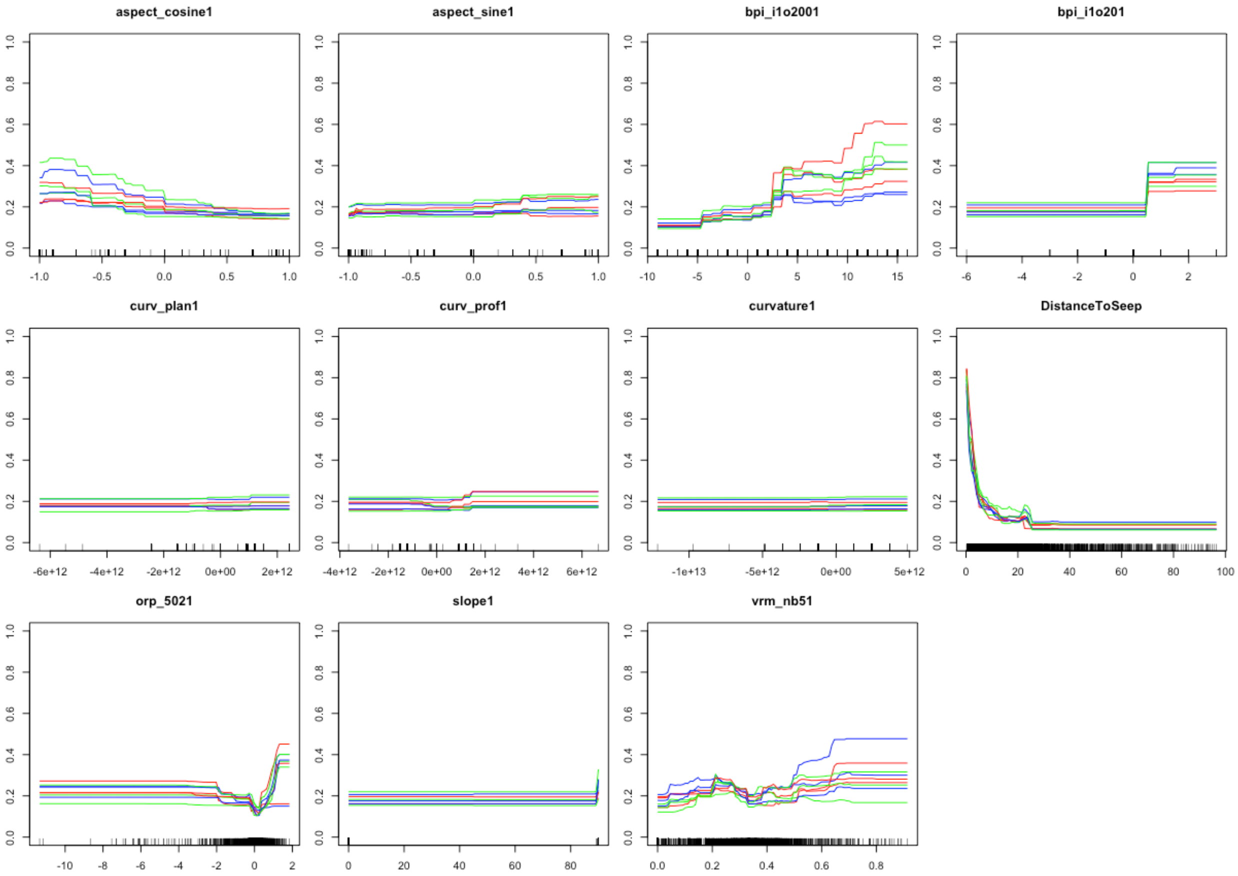

Figure S6. Response curves from HSMs - GBM

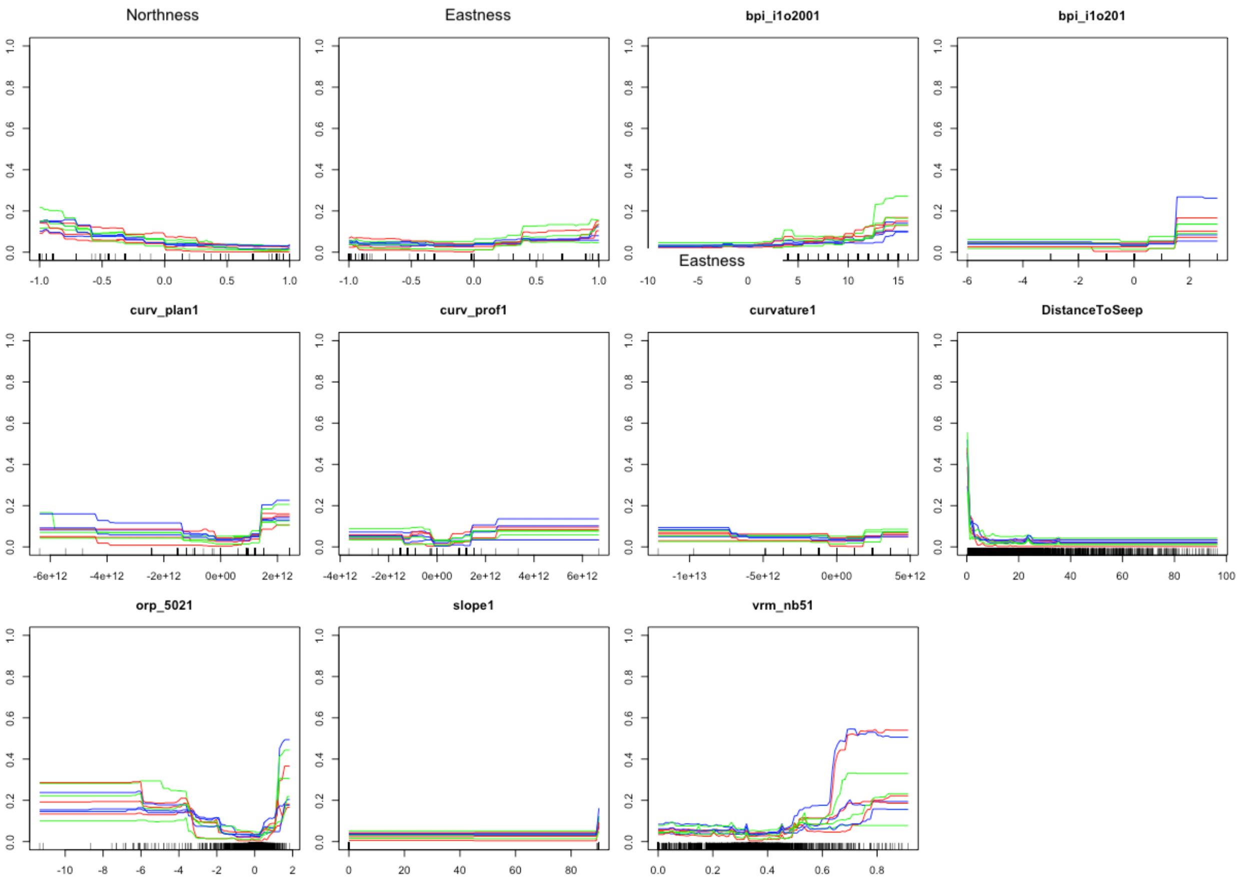

Figure S7. Response curves from HSMs - RF

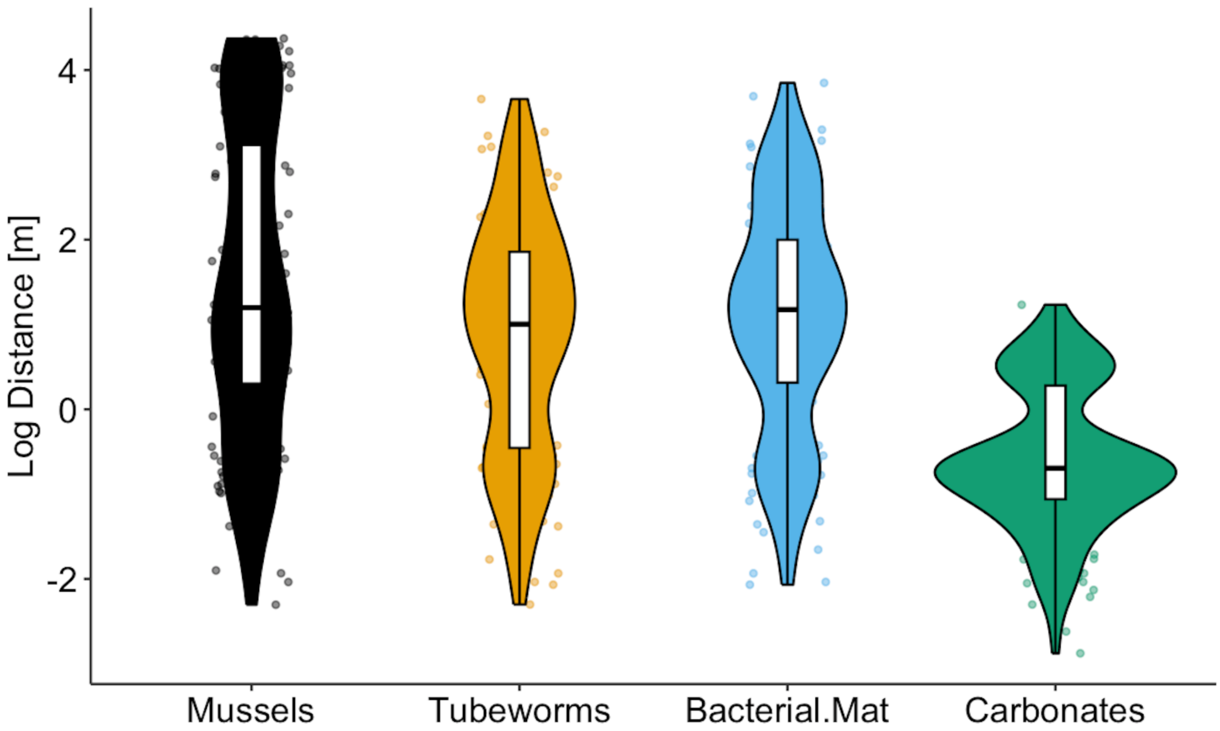

Figure S8**.** Violin plots showing the distribution of log-transformed distances (in meters) from *S. sahlingi* individuals to four seep indicators and substrate: Mussels, Tubeworms, Bacterial Mats, and Carbonates

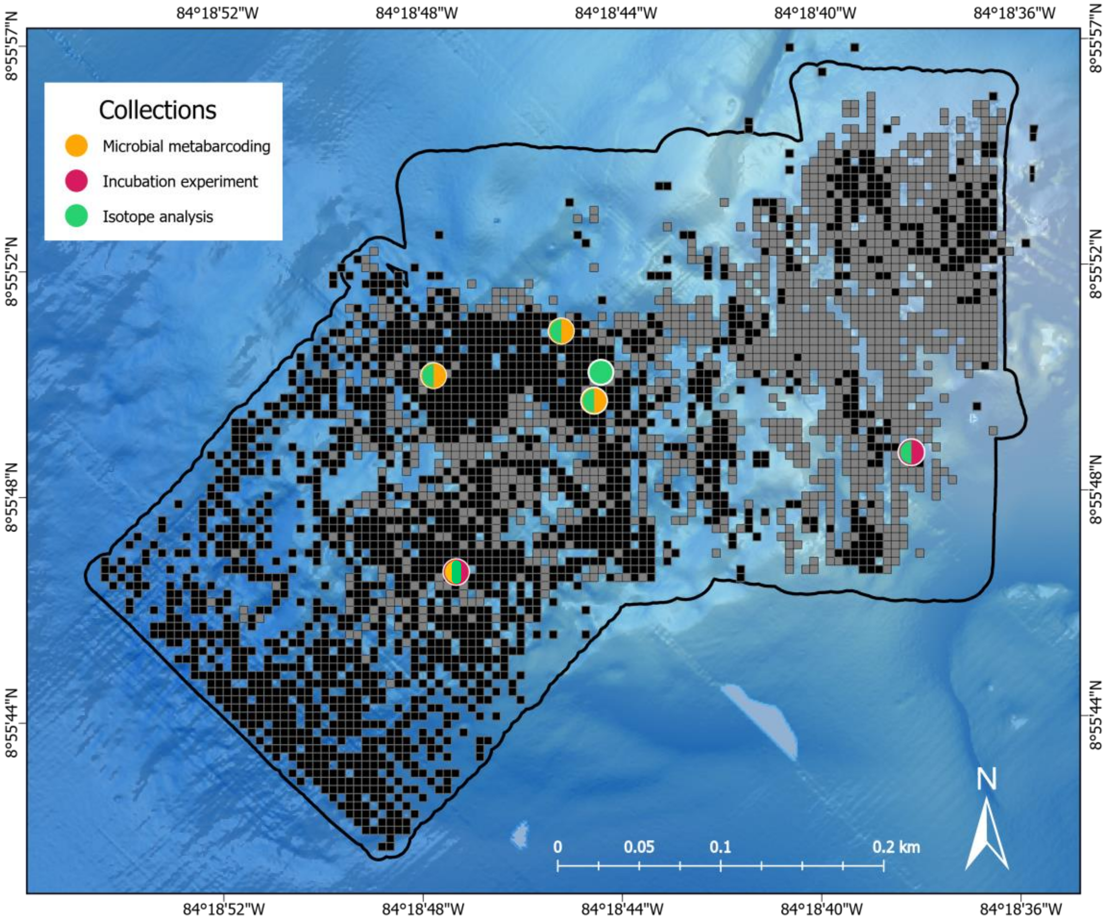

Figure S9. Sample collection locations at Mound 12. Symbols indicate locations where individual *Swiftia sahlingi* colonies were collected for microbial metabarcoding (orange), isotope incubation experiments (pink) and stable isotope analysis (green). Background grid cells show active (black) and transition (grey) areas.
